# Reflections on the first year of Plant Direct

**DOI:** 10.1002/pld3.98

**Published:** 2018-11-29

**Authors:** Ivan Baxter

We launched *Plant Direct* in March 2017 and published our first papers in July. Along the way, we worked out a lot of kinks in our process—this is the first journal launch I have been a part of, and I can honestly say that I never imagined how many little details you need to work out to make a journal run. Without the expert help from the sponsors Wiley and our founding societies—the American Society of Plant Biologists (ASPB) and the Society of Experimental Biology (SEB)—we would have been in real trouble. Since that time, we have published 77 papers providing a sound science outlet for more than 441 authors, whose publication charges have been used to support the activities of ASPB and SEB. Of those, 16 papers were transferred with review from our partner journals (*Plant Physiology, The Plant Journal,* and *The Plant Cell*), allowing us to make rapid decisions (less than a week) and using the labor of our reviewing committee more efficiently. As of October 15, those 77 papers had been downloaded 35,500 times. Overall, we are very happy with the start we have made; we feel that we have made good progress in fulfilling our mission to help our community by giving them an easier, faster way to get all of their science out.

As we moved into our second year, we began looking for ways to improve on what we did in the first year and add new features to help serve our community even better. We are excited to announce that we are now indexed in Scopus. We have begun a major effort to train and utilize new members of our community as reviewers at the journal. Our Associate Reviewer Board (ARB) initiative has two components: (a) identify community members who are experienced in reviewing but want to do more and get them in our system with tags to make it easier to identify them and (b) identify community members in need of training and give them training, including mentored reviewing of actual submissions. The response to this initiative has been incredibly positive and frankly overwhelming to the screening system we had set up. But I am happy to report that we have added 44 reviewers to our system and have begun training our first class of ARB members.

We have also expanded the type of manuscripts that we will accept. In addition to primary research reports, we will now publish white papers and workshop reports from our community members. The people who put these manuscripts together are working hard to improve our community, and we are happy to provide them an avenue to publish their work. We hope that these improvements will make *Plant Direct* an even better option for publishing and supporting our community and hope you will consider us for your next manuscript.

Finally, I want to thank so many people for their efforts to help *Plant Direct* over the first year—it is truly a community effort. Our authors and reviewers have given their time and put up with us as we got everything working. The team that made it happen: the editorial board, including the Supervising Editors, Feng Chen, Ana Margarida Fortes, Tracy Lawson, Hsou‐min Li, and Esther van der Knapp; the team at Wiley, including Shantaya Brinson, Rosie Trice, Jose Moreira, and Vicky Johnson; and the publications teams at our society partners: Neil Olszewski, Nancy Winchester, Crispin Taylor, and Martin Parry.

Looking forward to what the next year will bring.

